# Redox Imbalance in Chronic Inflammatory Diseases

**DOI:** 10.1155/2022/9813486

**Published:** 2022-04-08

**Authors:** Ziqing Li, Dongliang Xu, Xinhua Li, Yilun Deng, Chaohong Li

**Affiliations:** ^1^Department of Joint Surgery, Shandong Provincial Hospital Affiliated to Shandong First Medical University, Jinan, China; ^2^Orthopaedic Research Laboratory, Medical Science and Technology Innovation Center, Shandong First Medical University & Shandong Academy of Medical Sciences, Jinan, China; ^3^Department of Joint Surgery, The First Affiliated Hospital of Sun Yat-Sen University, Sun Yat-Sen University, Guangzhou, China; ^4^Department of Orthopedics, Shanghai General Hospital, Shanghai Jiao Tong University School of Medicine, Shanghai, China; ^5^Department of Medicine, Hematology & Oncology Division, University of Texas Health San Antonio, San Antonio, USA; ^6^Department of Histology and Embryology, Zhongshan School of Medicine, Sun Yat-Sen University, Guangzhou, China

Redox homeostasis, a crucial determinant of physiological processes that maintain the health of cellular function, is intimately relying on the balance between prooxidative productions and concomitant antioxidant defense system [1, 2]. Often caused by an aggressive accumulation of reactive oxygen species (ROS) or weakened antioxidant defenses in multiple organs, stubborn redox imbalance is one of the notorious features in patients suffering from chronic inflammatory diseases [2]. ROS are produced in all cell types as byproducts of the mitochondrial electron transport chain (ETC) during ATP synthesis or formed by NADPH oxidase (NOX) in a controlled manner for targeted purposes such as the respiratory burst in macrophages and neutrophils against microbial invasion [3]. In turn, organisms have evolved several defense mechanisms to detoxify ROS, ranging from transcription factors to enzymes that elevated intracellular antioxidative defenses [4, 5]. Also, many natural products and organic compounds demonstrate a prominent protective effect of antioxidants on targeted tissues, contributing to the relief of chronic inflammatory symptoms in patients [6]. Therefore, understanding the complicated relationship between redox regulation and concomitant inflammatory consequences, from a molecular basis to tissue levels, represents a significant challenge for developing efficient therapeutics for chronic inflammatory diseases. This special issue is aimed at presenting recent research efforts in both molecular and macro mechanisms of redox regulation during the chronic inflammatory process and the novel role of antioxidant factors in protecting organs from chronic inflammatory damage.

Bone marrow mesenchymal stem cell- (BMSC-) based therapy is a promising strategy for osteoporosis (OP) treatment. The study by J. Gao et al. reports Icariin (ICA), a traditional Chinese medicine that is used for tonifying the kidneys, could also promote the proliferation and osteogenic differentiation of BMSCs through the sclerostin/Wnt/*β*-catenin signaling pathway. By overexpressing or knocking down sclerostin gene in rat BMSCs, the authors found that sclerostin significantly inhibited BMSC proliferation and downregulated osteogenic genes (Runx2, *β*-catenin, and c-myc) and antioxidant factors (Prdx1, Cata, and Nqo1), whereas the presence of ICA could restore these inhibitory effects via the activation of Wnt/*β*-catenin pathway and the upregulation of antioxidant factors. Therefore, ICA may promote the therapeutic efficiency of BMSC-based regenerative therapy for OP.

Osteoarthritis (OA) is a debilitating disease and leads to chronic disability in old people, whereas total hip arthroplasty (THA) is an effective way for late-stage treatment. However, as a severe complication of THA, osteolysis leads to prosthesis loosening and additional revision surgery for OA patients. By using RNA sequencing, the study by G. Yang et al. explores the relevant molecular biomarkers for osteolysis after THA and identifies expressed mRNAs and lncRNAs during OA and osteolysis. The authors highlighted four identical interaction pairs, including two shared lncRNA-mRNA interaction pairs during OA and osteolysis (AC111000.4-CD8A and AC016831.6-CD8B) and other two osteolysis-specific interaction pairs (AC090607.4-FOXO3 and TAL1-BALON), contributing to the understanding of osteolysis pathophysiology.

Oxidative stress and microRNAs (miRNAs/miRs) affect the osteogenic induction of adipose-derived mesenchymal stem cells (ADSCs). The study by Y. Ye et al. investigates the mechanism of miR-125a-5p in regulating the osteogenesis of human ADSCs (hADSCs) under oxidative stress and finds that the expression of miR-125a-5p was negatively correlated with the osteogenic differentiation of hADSCs. The authors further demonstrated that miR-125a-5p was induced under oxidative stress and inhibited the expression of VEGF, leading to the reduction of osteogenic differentiation of hADSCs and proving to be a potential clinical target for bone repairing.

Temporomandibular joint osteoarthritis (TMJOA) is a typical chronic inflammatory disease characterized by the degradation of mandibular condylar cartilage (MCC). NF-*κ*B plays a crucial role in the inflammatory and immune responses during the development of TMJOA. The study by W. Li et al. investigates the protective effects of resveratrol (RES) against MCC degradation via antioxidant characteristics and regulation of COX-2/NF-*κ*B expression. The authors showed that RES could reverse the MCC degradation caused by inflammation. For the mechanism study, RES exerted the antioxidant effects by downregulating COX-2/NF-*κ*B/MMP expression and increasing cartilage markers, suggesting the therapeutic effects of RES against chondrocyte apoptosis of MCC during TMJOA.

Intervertebral disc degeneration (IVDD) is bound up with oxidative stress that caused intervertebral disc senescence. A clear diagnosis of IVDD at the early stage is pretty challenging. To assess the application value of serum cartilage oligomeric matrix protein (COMP) and extracellular matrix degradation products of C-telopeptide of type II collagen (CTX-II) as molecular markers for IVDD diagnosis, a comprehensive analysis based on protein expression, histological, and MRI changes in adult male rats during IVDD was carried out by D.-D. Qi et al. The authors reported that the expressions of COMP and CTX-II increased with the process of IVDD. Moreover, oxidative stress markers were found to correlate with CTX-II and COMP, wherein MDA (malondialdehyde) was positively correlated and SOD (superoxide dismutase) was negatively correlated with CTX-II and COMP, indicating reliable diagnostic markers for IVDD.

Promoting rehabilitation training in postoperative patients reduces overall oxidative stress caused by OA and surgical trauma. Compared with traditional oral education, video-assisted health education is believed to be an effective way in accelerating rehabilitation. By assessing clinical outcomes, inflammatory biomarkers, and monitoring rehabilitation progress of postoperative patients, P. Li et al. evaluate the effectiveness of video-assisted health education in promoting the postoperative rehabilitation of OA patients, as well as in reducing the job stress and burnout of participated nurses. The authors suggested that video-assisted health education could significantly promote patients' recovery after receiving total knee arthroplasty and reduce job stress in nurses when compared with traditional oral education.

Due to excellent characteristics (high efficiency, sustained stability, and low costs) compared to natural enzymes, nanozymes have been widely studied and applied in various fields such as clinical medicine, basic science, chemical engineering, food industry, and even agriculture. The review by H. Wang et al. summarizes the utilization development of nanozymes in the medicine field and discusses the therapeutic applications of antioxidant-like properties of nanozymes in treating chronic inflammatory diseases. In addition, multiple materials and different types of nanozymes that are used for the treatment of chronic inflammatory diseases were also summarized in this review.

In conclusion, a comprehensive understanding of ongoing efforts would help researchers to identify efficient approaches in the field and eventually lead to the successful discovery of therapeutics. The guest editorial team wishes that this special issue will help in evidencing research from multiple disciplines in this area and encourage future collaborations from multidisciplinary aspects.
